# Genomic Analysis Provides Insights Into the Plant Architecture Variations in *in situ* Conserved Chinese Wild Rice (*Oryza rufipogon* Griff.)

**DOI:** 10.3389/fpls.2022.921349

**Published:** 2022-06-27

**Authors:** Ziyi Yang, Yilin Zhang, Meng Xing, Xiaowen Wang, Zhijian Xu, Jingfen Huang, Yanyan Wang, Fei Li, Yamin Nie, Jinyue Ge, Danjing Lou, Ziran Liu, Zhenyun Han, Yuntao Liang, Xiaoming Zheng, Qingwen Yang, Hang He, Weihua Qiao

**Affiliations:** ^1^Institute of Crop Sciences, Chinese Academy of Agricultural Sciences, Beijing, China; ^2^National Nanfan Research Institute, Chinese Academy of Agricultural Sciences, Sanya, China; ^3^State Key Laboratory of Protein and Plant Gene Research, School of Advanced Agriculture Sciences and School of Life Sciences, Peking University, Beijing, China; ^4^Rice Research Institute, Guangxi Academy of Agricultural Sciences, Nanning, China; ^5^Institute of Advanced Agricultural Sciences, Peking University, Weifang, China

**Keywords:** Chinese wild rice, *in situ* conservation, genomic analysis, plant architecture variation, *OsDHD1*

## Abstract

*In situ* conserved wild rice (*Oryza rufipogon* Griff.) is a promising source of alleles for improving rice production worldwide. In this study, we conducted a genomic analysis of an *in situ* conserved wild rice population (Guiping wild rice) growing at the center of wild rice genetic diversity in South China. Differences in the plant architecture in this population were investigated. An analysis using molecular markers revealed the substantial genetic diversity in this population, which was divided into subgroups according to the plant architecture. After resequencing representative individuals, the Guiping wild rice population was compared with other *O. rufipogon* and *Oryza sativa* populations. The results indicated that this *in situ* conserved wild rice population has a unique genetic structure, with genes that were introgressed from aromatic and *O. sativa* ssp. *indica* and *japonica* populations. The QTLs associated with plant architecture in this population were detected *via* a pair-wise comparison analysis of the sequencing data for multiple DNA pools. The results suggested that a heading date-related gene (*DHD1*) might be associated with variations in plant architecture and may have originated in cultivated rice. Our findings provide researchers with useful insights for future genomic analyses of *in situ* conserved wild rice populations.

## Introduction

Asian cultivated rice (*Oryza sativa*) is one of the oldest staple food crops widely consumed by half of the global population, which is expected to grow by 25% and reach 10 billion over the next 30 years. Developing new rice varieties that are higher yielding and more nutritious than current cultivated varieties as well as resistant to pests and diseases and ideal for climate-smart agricultural production is critical for avoiding widespread malnutrition or starvation (Lee et al., 2019). Although the rice domestication history remains controversial, rice is thought to have been domesticated from the wild relative *Oryza rufipogon* in China approximately 9,000 years ago (Oka, 1988; Fuller et al., 2010; Civan et al., 2015; Wang et al., 2017). *Oryza rufipogon* grows in pantropical regions, including the diverse natural habitats in South China (Gao et al., 1996; Gao, 2004). Additionally, wild rice can be crossed directly with cultivated rice because they have the same AA genome, making *O. rufipogon* a suitable genetic resource for enhancing rice characteristics. A key research milestone was the identification of the cytoplasmic male sterility genes in *O. rufipogon*. This enabled the development of three-line hybrid rice, which is a notable achievement in China, with worldwide implications.

China has one of the most abundant *O. rufipogon* resources worldwide. Specifically, *O. rufipogon* plants can be found in seven provinces and/or autonomous regions in China (i.e., Guangdong, Guangxi, Hainan, Yunnan, Hunan, Jiangxi, and Fujian), but their continued growth is at risk because of recent human activities and natural degradation (Xu et al., 2020). The *in situ* conservation of wild relatives of crops has been one of the key strategies for conserving biological diversity in China and elsewhere. Globally, China is the first country to implement the *in situ* conservation of wild rice. In the past two decades, there has been substantial progress in the *in situ* conservation of wild rice throughout China. Most of the endangered wild rice populations have been protected by both *in situ* and *ex situ* conservation programs (Xu et al., 2020). By the end of 2020, more than 30 national *in situ* conservation sites were constructed for wild rice in China (Yang et al., 2022). There is an urgent need for research that attempts to address many basic issues, including how to better develop and evaluate *in situ* conservation methods. Various molecular markers, such as simple sequence repeats (SSR) and nuclear intron targeting (InDel), were used to evaluate the genetic diversity in different crop wild relative species (Ramakrishnan et al., 2016). Research on the genetic diversity and population variation within a species may help clarify evolutionary processes and mechanisms, while also generating relevant information for designing appropriate and efficient *in situ* conservation strategies. However, analyses of genes and their introgression in or between *in situ* conserved populations remain limited.

The change from the creeping growth of cultivated rice ancestors to the erect growth of modern rice varieties is an example of the effect of domestication on plant architecture (Jin et al., 2008). Many plant architecture-related genes, such as *PROG1* and *LAZY1*, reportedly originated in *O. rufipogon* and have undergone natural variations in cultivated rice (Li et al., 2007; Tan et al., 2008). Most *O. rufipogon* plants exhibit creeping growth, but there are also some tilted and semi-erect types in some populations. In contrast, erect plants are rare in *O. rufipogon* populations. It is unclear whether erect wild rice plants are the result of the introgression of a gene from cultivated rice or a natural variation during the adaptation to environmental conditions.

In this study, we performed an extensive field and genomic investigation of a typical natural *O. rufipogon* population and its habitats within the *in situ* conservation sites at the center of wild rice genetic diversity in China. The plants in the selected population varied considerably in terms of plant architecture. The objectives of this study were as follows: (1) assess the genetic diversity within an *in situ* conserved population; (2) detect genes that were introgressed from other wild rice populations or local landraces; and (3) identify natural variations associated with plant architecture in the *in situ* conserved population. Our results provide useful information regarding plant architectural diversity and new insights for optimizing the *in situ* conservation of wild rice in China.

## Materials and Methods

### Population Sampling

To ensure the collected samples fully represented the genetic diversity of the population and to avoid collecting clones of the same individual, irregular grid lines were applied. Specifically, using an individual collection distance of about 5 m, a total of 184 randomly selected leaves were collected from their original habitat, Guiping *in situ* conservation site, placed in a plastic bag, and stored at –20°C. Collection of 30 samples in the mixing pool were performed also using a 5 m-distance rule. For the 20 re-sequenced samples, including 3 erect types samples, 4 semi-erect samples, 5 tilt samples, and 8 creep samples, same type individual collection distance of more than 20 m.

### DNA Isolation and Polymerase Chain Reaction

The collected leaves were ground to a powder in liquid nitrogen using the SPEX Sample Prep Geno/Grinder. Genomic DNA was extracted according to the CTAB method (Edwards et al., 1991). A PCR amplification was completed in a 15-μL reaction volume that comprised 4.3 μL ddH_2_O, 0.6 μL forward and reverse primers, 2 μL DNA, and 7.5 μL 2× Es Taq MasterMix. The PCR program was as follows: 95°C for 5 min; 35 cycles of 94°C for 30 s, 57°C for 30 s, and 72°C for 30 s; and 72°C for 2 min. The amplified products were separated in 6% denaturing polyacrylamide gels.

### Statistical Analysis

The simple sequence repeat (SSR) data format was converted to readable input files for STRUCTURE, PowerMarker, and POPGENE. We used POPGENE 1.31 (Yeh et al., 1998) to calculate the following genetic diversity-related parameters: Shannon–Weaver information index (*I*), observed heterozygosity (*He*), and expected heterozygosity (*Ho*) (Nei, 1978). STRUCTURE was used to infer genetic clustering (K) according to a model-based clustering method (Falush and Pritchard, 2003). We evaluated K values from 2 to 9 by performing 10 independent runs for each K value; the model was repeated with a 10,000 burn-in period and 100,000 Monte Carlo Markov chain repetitions. CLUMPP (version 1.1) (Jakobsson and Rosenberg, 2007) was used to obtain the best clustering for each K value. The unweighted pair group method with arithmetic mean (UPGMA) (Liu and Muse, 2005) of PowerMarker was used to evaluate the relationships between populations according to Nei’s standard genetic distance (Nei, 1972).

Sequences were aligned using the MUSCLE program in MEGA 7. A phylogenetic analysis involving nucleotide sequences was conducted using the maximum likelihood method, with 1,000 bootstrap replicates. A model-based estimation of the ancestry coefficient was performed using ADMIXTURE (Alexander et al., 2009). High-quality variations, including single nucleotide polymorphisms (SNPs) and insertions/deletions (InDels), were identified by analyzing VCF files according to the following four criteria: (1) maximum missing rate < 0.2; (2) minimum read depth of 5; (3) biallelic position; and (4) genotype quality > 20. A haplotype network was constructed using the minimum spanning method implemented in POPART.

### Pair-Wise Comparison Analysis for Multiple Pool-Seq (PCAMP)

We developed four DNA bulks from erect, semi-erect, tilted, and creeping Guiping plants at the *in situ* conservation site. The sample detection, library construction, library quality detection, and onboard sequencing were performed according to the standard Illumina protocols. After confirming the quality of the genomic DNA was sufficient for the subsequent analysis, the DNA was fragmented *via* a mechanical method. The ends of the fragmented DNA were repaired and a poly-A tail was added to the 3’ end. After adding a sequencing adapter, a PCR amplification was performed and the resulting products were purified to complete the construction of the sequencing library. The high-quality library was sequenced using an Illumina system. We used the BWA software to align the short reads of the DNA bulks to the Nipponbare reference genome (MSU_v7.0) (Li and Durbin, 2009). The GATK software toolkit was used to detect SNPs (Yang et al., 2019). On the basis of the localization of the clean reads in the reference genome, Picard was used to eliminate redundant reads. The SnpEff software (Cingolani et al., 2012) was used to annotate mutations (SNPs and small InDels) and predict the effects of the mutations. We calculated the Euclidean distance (ED) to identify the candidate regions associated with plant architecture. The ED was calculated using the following formula:


$$E\,D=\sqrt{\left(A\,m\,u\,t-A\,w\,t\right)\,2+\left(C\,m\,u\,t-C\,w\,t\right)\,2+}\,\sqrt{\left(G\,m\,u\,t-G\,w\,t\right)\,2+\left(T\,m\,u\,t-T\,w\,t\right)\,2},$$

where A_*mut*_, C_*mut*_, G_*mut*_, and T_*mut*_, respectively represent the frequency of A, C, G, and T bases in the mixed mutant pool, whereas A_*wt*_, C_*wt*_, G_*wt*_, and T_*wt*_ respectively represent the frequency of A, C, G, and T bases in the mixed wild-type pool. During the analysis, there was a SNP site that differed between the two mixed genotypes. Additionally, the depth of the individual bases in the different mixed pools was statistically significant and the ED value for each site was calculated. The background noise was eliminated by applying the 5th power of the original ED as the correlation value (Hill et al., 2013). The distance method was then used to fit the ED value. The median + three standard deviations of the fitted values for all loci was set as the correlation threshold (Hill et al., 2013) to identify candidate regions. Using InDels for the association analysis, which was performed according to the method used for the SNP association analysis, the final candidate regions were identified by determining the intersecting regions corresponding to SNPs and InDels.

## Results

### Analysis of the Genetic Diversity of an *in situ* Conserved Wild Rice Population

**FIGURE 1:**
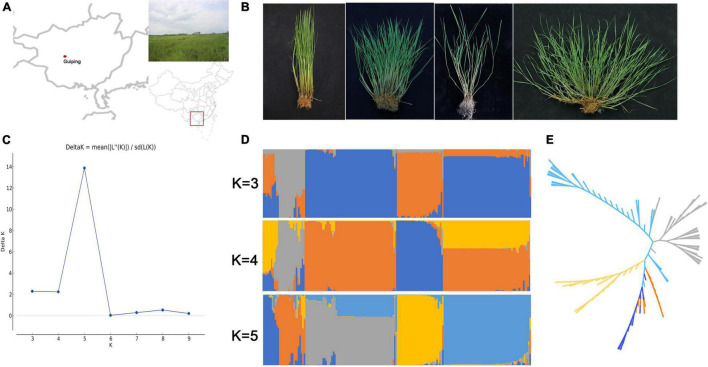
Genetic diversity in the Guiping *in situ* conserved wild rice population. **(A)** Location of the Guiping *in situ* wild rice conservation site. **(B)** Four plant architecture types among the *Oryza rufipogon* individuals in the Guiping population (left to right: erect, semi-erect, tilted, and creeping). **(C)** Delta K values for the STRUCTURE analysis. **(D)** Clustering of 184 individuals in the Guiping wild rice population according to the STRUCTURE analysis (*K* = 3–5). **(E)** UPGMA dendrograms constructed on the basis of Nei’s standard genetic distance.

The *in situ* conservation site for wild rice in this study was located in Guiping county, Guangxi province, China (N 22°52′, E 109°41′) (Figure 1A). This site includes a typical natural *O. rufipogon* population. First, it is located at the center of wild rice genetic diversity in China. Second, this population is the biggest natural *O. rufipogon* population worldwide. This *in situ* conservation site, which was constructed in 2001, comprises 4.21 ha with 2.6 million *O. rufipogon* plants (Xu et al., 2020). An examination of plant architecture revealed the following four plant types: erect, semi-erect, tilted, and creeping (Figure 1B), which accounted for 1.9, 2.5, 4.4, and 91.2% of the whole population, respectively.

The genetic diversity of this population was determined by analyzing 184 individual plants. More than 80 SSR and InDel primer pairs stored in our laboratory were included in the analysis. Finally, 16 SSR markers and 24 InDel markers that were polymorphic and evenly disturbed on 12 chromosomes were selected for further study (Supplementary Table 1). The mean values for *Ae* (effective number of alleles), *He*, *Ho*, and *I* across all loci were 1.204, 0.852, 0.148, and 0.259, respectively (Table 1). The genetic relationships among samples were analyzed using STRUCTURE and the constructed UPGMA dendrogram. For the structural analysis, when the population number K was 5, the log likelihood ln[P(D)] was highest (Figure 1C). Accordingly, five groups were determined (Figure 1D). All erect and semi-erect individuals were classified in Group 2, whereas tilted individuals were classified in Groups 1 and 2 and creeping individuals were classified in all groups except for Group 2, although they were mainly in Groups 3–5. The UPGMA dendrogram revealed five main clusters, with similar structural results (Figure 1E).

**TABLE 1:** Genetic diversity-related parameters for different SSR and InDel loci.

Locus	Chromosome	He	Ho	Nei	Ae	I
Indel1-4	1	0.859	0.141	0.1406	1.1636	0.2691
Indel1-9	1	0.9892	0.0108	0.0108	1.0109	0.0338
RM10716	1	0.8727	0.1273	0.127	1.15	0.2428
Indel2-4	2	0.8498	0.1502	0.1498	1.1761	0.2825
Indel2-3	2	0.8205	0.1796	0.1791	1.2432	0.3066
RM12923	2	0.8518	0.1482	0.1478	1.1978	0.2559
RM13406	2	0.5024	0.4976	0.4962	1.985	0.6894
Indel3-7	3	0.8835	0.1165	0.1162	1.1371	0.225
Indel3-23	3	0.9784	0.0216	0.0215	1.022	0.06
RM14759	3	0.8408	0.1592	0.1588	1.1888	0.2954
RM15347	3	0.5982	0.4018	0.4007	1.6686	0.5903
Indel4-3	4	0.8683	0.1317	0.1313	1.1512	0.2553
Indel4-10	4	0.9784	0.0216	0.0215	1.022	0.06
RM16555	4	0.9053	0.0947	0.0945	1.1083	0.1915
RM5424	4	0.8057	0.1943	0.1938	1.2403	0.3438
Indel5-2	5	0.9784	0.0216	0.0215	1.022	0.06
Indel5-9	5	0.7972	0.2028	0.2022	1.2535	0.3551
RM18502	5	0.8683	0.1317	0.1313	1.1512	0.2553
Indel6-4	6	0.8498	0.1502	0.1498	1.1761	0.2825
Indel6-9	6	0.8748	0.1252	0.1249	1.1467	0.241
RM19725	6	0.9367	0.0633	0.0631	1.0673	0.1437
RM20111	6	0.9784	0.0216	0.0215	1.022	0.06
Indel7-5	7	0.8057	0.1943	0.1938	1.2403	0.3438
Indel7-9	7	0.8173	0.1827	0.1822	1.2493	0.3112
RM21236	7	0.8408	0.1592	0.1588	1.1888	0.2954
Indel8-3	8	0.9784	0.0216	0.0215	1.022	0.06
Indel8-8	8	0.947	0.053	0.0529	1.0558	0.1248
RM22959	8	0.9784	0.0216	0.0215	1.022	0.06
Indel9-2	9	0.8683	0.1317	0.1313	1.1512	0.2553
Indel9-8	9	0.9241	0.0759	0.0757	1.0873	0.1515
RM23842	9	0.6531	0.3469	0.3459	1.5547	0.5255
Indel10-6	10	0.7807	0.2193	0.2188	1.28	0.3768
Indel10-8	10	0.9626	0.0374	0.0373	1.0388	0.094
RM25375	10	0.6698	0.3302	0.3293	1.5197	0.5051
Indel11-4	11	0.7461	0.2539	0.2533	1.3924	0.3944
Indel11-9	11	0.8873	0.1127	0.1124	1.1267	0.2264
RM26319	11	0.8407	0.1593	0.1589	1.2266	0.2675
Indel12-3	12	0.8172	0.1828	0.1823	1.2297	0.3242
Indel12-9	12	0.9383	0.0618	0.0616	1.0688	0.1301
RM28107	12	0.7279	0.2721	0.2714	1.4008	0.4352
Mean		0.8518	0.1482	0.1478	1.204	0.2595

### Comparative Analysis of the Guiping Wild Rice and Cultivated Rice Genomes

**FIGURE 2:**
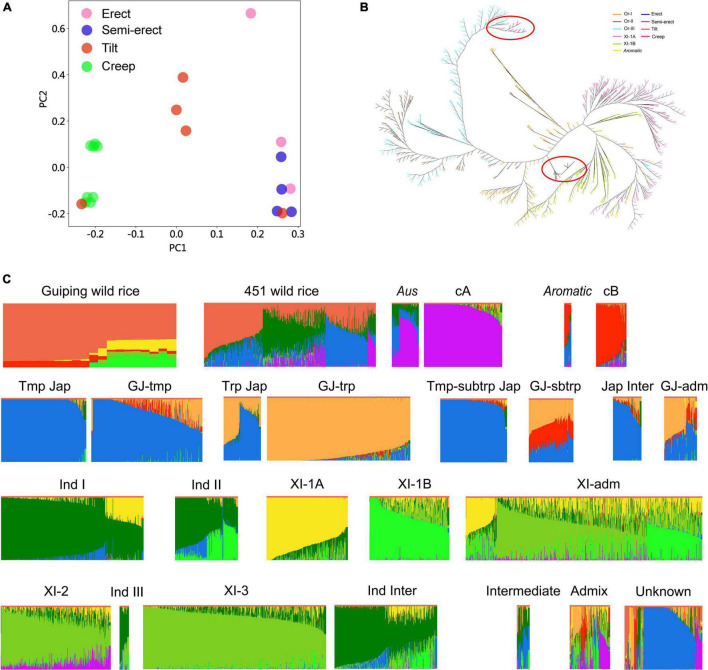
Genetic structure and association analyses of the Guiping wild rice population. **(A)** Principal component analysis of 20 individuals from the Guiping wild rice population. **(B)** Neighbor-joining tree of the Guiping wild rice population, 451 *O. rufipogon* accessions, 209 XI-1A accessions, 205 XI-1B, and 96 *aromatic* accessions analyzed in an earlier study (Huang et al., 2012). On the basis of approximately 5 million SNPs, the following groups were identified: Or-I (orange), Or-II (brown), Or-III (light blue), XI-1A (pink), XI-1B (green), *aromatic* (yellow), erect (dark blue), semi-erect (purple), tilted (red), and creeping (rose red). Guiping wild rice were marked with red cycles. **(C)** Ancestry of the 20 individuals from the Guiping wild rice population. Genomic data for 451 wild rice accessions were obtained from a published study (Huang et al., 2012). Data for the other 5,152 cultivated rice accessions were downloaded from the Genome Variation Map database (see footnote 1). cA, *Aus*; cB, Aromatic; Tmp jap, Temperate–*Japonica*; Trp jap, Tropical–*Japonica*; Tmp-subtrp jap, Temperate subtropical–*Japonica*; GJ-tmp, East Asian temperate–*Japonica*; GJ-subtrp, Southeast Asian subtropical–*Japonica*; GJ-trp, Southeast Asian tropical–*Japonica*; Jap inter, *Japonica*–Intermediate; Ind I, *Indica* I; XI-1A, East Asia *Indica*; Ind II, *Indica* II; XI-1B, Diverse origins–*Indica*; XI-adm and GJ-adm, Accessions with admixture components <0.65 within XI and GJ were classified as “XI-adm” and “GJ-adm”; Ind Inter, *Indica*–Intermediate; Ind III, *Indica* III; XI-2, South Asia–*Indica*; XI-3, Southeast Asia–*Indica*; Intermediate; Admix, Accessions between major groups; Unknown.

Twenty *O. rufipogon* accessions in the *in situ* conservation site were selected for genome sequencing (i.e., three erect, four semi-erect, five tilted, and eight creeping accessions). After aligning the reads to the rice reference genome sequence, we identified 5,037,497 non-singleton SNPs. On the basis of the SNP data, the sequence diversity (π) of *O. rufipogon* was estimated to be about 0.003, which is higher than that of *O. sativa* (0.0024), *O. sativa* ssp. *indica* (0.0016), and *O. sativa* ssp. *japonica* (0.0006) (Huang et al., 2012). A principal component analysis (PCA) indicated that these accessions could be grouped according to their plant architecture (Figure 2A). We also investigated the population structure of 451 wild rice accessions that were included in an earlier study (Huang et al., 2012). Interestingly, the neighbor-joining tree showed that the creep and most tilt phenotypes of Guiping wild rice comprises Or-III plants, most of which originated in Guangxi province. While erect and semi-erect wild rice clustered in Or-I, which clustered closer to *indica* (Figure 2B).

To compare our Guiping wild rice population with worldwide wild and cultivated rice populations, the whole-genome sequencing data for 5,152 cultivated rice accessions (5K database) were downloaded from the Genome Variation Map database.^1^ We compared our data with the 5K data and the published data for 451 wild rice accessions (Huang et al., 2012). Germplasm groups were defined by ADMIXTURE (Alexander et al., 2009), with the number of ancestral populations (K) ranging from 6 to 9 (Figure 2C and Supplementary Figure 1). The results indicated that most of the genetic components in the 20 Guiping wild rice accessions were from wild rice, but some were from Aromatic, XI-1A, and XI-1B (*K* = 9, Figure 2C). They were divided into the following nine groups: *Indica*, *Japonica*, Tropical–*Japonica*, Temperate–*Japonica*, *O. rufipogon*, *Aus*, *Aromatic*, Intermediate-type, and Other. An examination of the Guiping wild rice accessions with erect or semi-erect phenotypes revealed their genetic components were mainly from wild rice (about 60%), XI-1B (about 20%), and XI-1A (about 15%), but a small percentage was from Aromatic. In contrast, the genetic components of the accessions with a tilted phenotype were primarily from wild rice (about 80%) and Aromatic (about 10%), but a small proportion was from XI-1A. The genetic components of the accessions with a creeping phenotype were mostly from wild rice (about 90%), with approximately 10% from Aromatic. The ABBA results also indicated that the 20 Guiping wild rice accessions had genetic components from Aromatic, XI-1A, and XI-1B (Supplementary Table 2).

### Identification of Main-Effect Variations in the Plant Architecture of Guiping Wild Rice

A PCAMP protocol (Yang et al., 2019) was used to identify candidate genomic regions related to plant architecture in the wild rice population. The whole population was divided into the following four subpopulations that differed in terms of plant architecture: R01-erect, R02-semi-erect, R03-tilted, and R04-creeping. Genomic DNA was extracted from 30 phenotypically identical individuals from each subpopulation and then combined as one bulk for the pair-wise comparison. The R01, R02, R03, and R04 mixed DNA was sequenced using the Illumina HiSeq X 10 high-throughput sequencing platform. After filtering the raw data, 85.18 Gb remained, of which R01, R02, R03, and R04 accounted for 13.81, 17.29, 20.75, and 34.33 Gb, respectively. The average sequencing depth was 48.5-fold, covering 97.76% of the whole genome.

**FIGURE 3:**
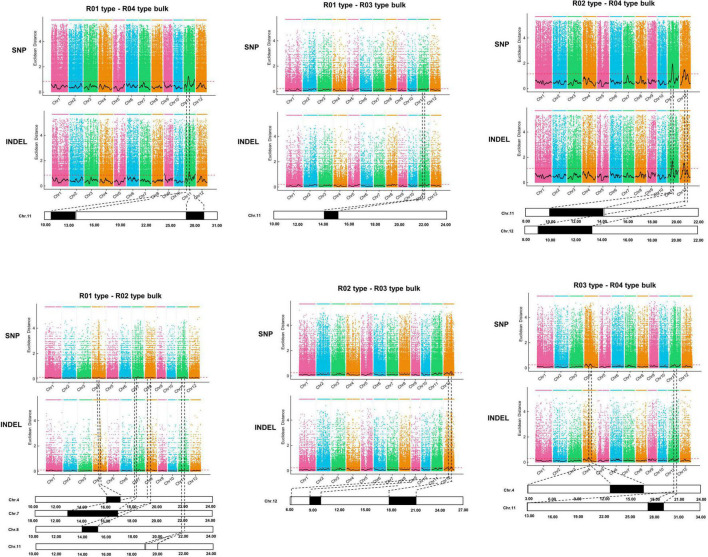
Identification of genomic regions controlling plant architecture in the Guiping wild rice population according to the PCAMP data. Four plant architecture types: R01-erect, R02-semi-erect, R03-tilted, and R04-creeping. Thirty individuals were selected for each type to construct a mixed DNA pool. A bulked segregant analysis was performed for each type. The Euclidean distance (ED) value of SNPs and InDels was calculated to identify the candidate regions for each comparison. The overlapping physical positions on the same chromosome for each comparison are in black.

**FIGURE 4:**
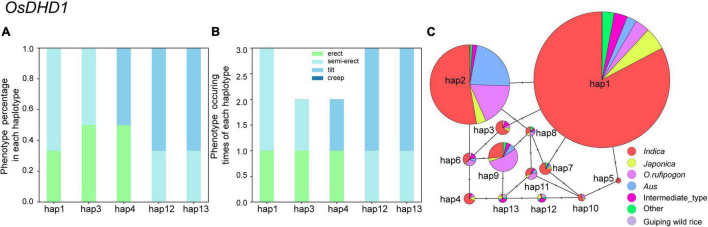
Analysis of the haplotype networks of *OsDHD1* in the Guiping wild rice population and the 5K dataset. **(A,B)** Phenotypic analysis according to the *OsDHD1* haplotype. **(C)** Haplotype networks of *OsDHD1*. The circle size is proportional to the sample quantity within a given haplotype. The short solid lines represent genetic distances between haplotypes.

To identify candidate genomic regions responsible for the plant architecture of the Guiping wild rice population, we compared the SNPs and InDels between DNA pools. We used ED-based methods involving SNPs and InDels to identify candidate regions. The overlapping physical positions on the same chromosome were selected in each comparison (Figure 3). The intersecting regions (Table 2) were selected as the final candidate regions that were significantly associated with plant architecture-related genes in the wild rice population. A *DHD1* gene on chromosome 11 had the highest ED value in the R01–R04 comparison. In a recent study, *DHD1* was revealed to regulate the rice heading date, while also influencing the plant architecture (Zhang et al., 2019). No other reported plant architecture-related genes were detected in these overlapping regions. Hence, *DHD1* might be the candidate gene mediating the variations in the plant architecture in the Guiping wild rice population. To confirm this possibility, we analyzed the following 10 well-documented plant architecture-related genes: *OsPROG1*, *OsLAZY1*, *OsMOC1*, *OsTAC1*, *OsLIC1*, *OsDLT*, *OsMOC3*, *OsTB1*, *OsTAD1*, and *OsDHD1* (Supplementary Table 3). Using data for our 20 Guiping wild rice accessions and 451 *O. rufipogon* accessions as well as the data in the 5K database, the genic and 1-kb flanking regions of these 10 genes were used for a variant analysis. Their haplotype networks were constructed according to the minimum spanning method implemented in POPART. Notably, Only *DHD1* had a unique haplotype related to the erect/semi-erect phenotype in the Guiping wild rice population. Other genes, including *OsPROG1*, *OsLAZY1*, and *OsMOC1* (Li et al., 2003; Li et al., 2007; Tan et al., 2008), which significantly influence rice plant architecture, had no erect phenotype-related haplotypes in the Guiping wild rice population (Supplementary Figures 2, 3). Thirty haplotypes were detected for *OsDHD1*, of which haplotypes 1, 3, and 4 were associated with the erect or semi-erect phenotype (Figure 4). The cultivated rice accessions with these haplotypes were mainly *indica* accessions belonging to Subgroup XI-A from East Asian countries, among them more than 52% (1408/2962) accessions were from China.

**TABLE 2:** Final genomic candidate regions related to the plant architecture in the Guiping wild rice population.

Chromosome	Genomic candidate regions (Mb)	Known genes
4	16.09–17.18	
8	14.27–15.43	
11	11.11–11.28	
11	27.89–29.01	*DHD1*
12	9.62–10.41	

## Discussion

*Oryza rufipogon* is an important germplasm resource for food security, but it is seriously endangered worldwide. Almost all studies on the *in situ* conservation of wild rice have been conducted in China. Most of these studies focused on the related policy, techniques, and methods for establishing *in situ* conservation sites. Thus, there are relatively few reports describing genetic analyses of the introgression involving other populations, including cultivated rice and other wild rice accessions.

In this study, a typical *in situ* conserved Chinese wild rice population in Guiping county of Guangxi province was investigated. The genetic diversity was first elucidated on the basis of 40 molecular markers (Supplementary Table 1). Compared with the genetic diversity detected in previous studies, the genetic diversity of the *in situ* conserved Guiping population was relatively stable. For example, Zhou et al. (2003) detected 12 populations in Guangxi, Gao (2004) analyzed six wild rice populations across China, and Yu et al. (2004) investigated the genetic diversity of *O. rufipogon* in many regions in Guangxi. These studies included the Guiping population, but samples were derived from germplasm (seed) banks or gardens (i.e., *ex situ* conservation sites). The genetic diversity indictors *Ho* and *I* in the current study were consistent with the corresponding values in these earlier studies. Considering the materials from *ex situ* conservation sites were collected from their original habitats 10 or 20 years ago, the *in situ* conservation site in Guiping was suitable for maintaining wild rice genetic diversity. Moreover, the sequence diversity (π) determined according to the SNP data (about 0.003) was similar to that of wild rice and much higher than that of cultivated rice in an earlier study (Huang et al., 2012). Accordingly, at the center of wild rice genetic diversity in China, the Guiping wild rice population is highly genetically diverse.

The central goal of the *in situ* conservation of wild rice is to prevent human activities from affecting genetic diversity and cultivated rice gene flow. We re-sequenced 20 individuals from this conservation site that varied in terms of plant architecture (Figure 1B). The PCA results implied that these 20 individuals could be grouped according to their plant architecture (Figure 2A). Additionally, the analysis of genetic structures involving SSR/InDel markers indicated that this population can be divided into five subpopulations (Figure 1D). These results revealed that the *in situ* conserved wild rice population in Guiping has a special plant architecture-related population structure. On the basis of published data (Huang et al., 2012), all of the samples collected from Guiping in this study were identified as Or-III plants (Figure 2B), indicative of a strong correlation with geographic distribution. The introgression between wild rice and XI-1A or XI-1B was also confirmed in an earlier investigation (Huang et al., 2012). However, there are no reports suggesting that wild rice has genetic components from aromatic rice. In the current study, we detected genes in the Guiping wild rice genome that were introgressed from aromatic rice populations. Moreover, ABBA–BABA data also suggested that the Guiping wild rice genome includes genes from *indica* rice (Supplementary Table 2).

The Guiping wild rice population, which is the largest *in situ* conserved wild rice population, grows in the central Pearl River region, which is where rice was first domesticated (Huang et al., 2012). There were no plants with an erect phenotype when the conservation site was established 20 years ago. To clarify how variations in plant architecture developed, we identified the main-effect variants using a PCAMP approach and constructed haplotype networks for 10 plant architecture-related genes. Introgressions were revealed only for *OsDHD1*. The PCAMP results also suggested that *OsDHD1* may be a candidate gene associated with plant type variations. We also detected variations in major plant architecture-related genes, such as *PROG1* and *LAZY1*, in the wild rice population, but they did not affect plant architecture. An examination of the cultivated rice accessions that were the source of the introgressed genes in Guiping wild rice revealed a lack of accessions in Guiping with *OsDHD1* haplotypes 1, 3, or 4, but a few cultivated rice varieties or landraces native to Guangxi province with these haplotypes were identified.

Because only growing closely related *Oryza* species may result in wild rice pollen infiltration, we speculated that the plant architecture variations may have been caused by the introgression of genes from Guiping landraces, but also by the infiltration of pollen from major commercially cultivated rice varieties. A good plant structure may positively affect the planting density and photosynthetic efficiency, while also providing plants with a competitive advantage. Therefore, variations in plant architecture may be the result of natural genomic modifications that enable adaptations to environmental conditions. The Guiping *in situ* wild rice conservation site has metal fences to keep out livestock and is surrounded by many fields containing cultivated rice varieties. Accordingly, we strongly recommend that the *in situ* conservation site should be surrounded by a fence that can appropriately isolate the area and prevent the infiltration of alien pollen. Furthermore, in future related genomic investigations, additional individuals should be collected for *ex situ* conservation, identification, and evaluation to conserve the genetic integrity of the population and to identify elite genes in the population relevant for improving rice production.

## Data Availability Statement

The data presented in this study are deposited in NCBI repository, accession number: SRR19425523–SRR19425542.

## Author Contributions

ZY and YZ performed most of the experiments, including phenotyping, data analysis, and haplotype analysis. YZ, MX, and XW participated in the genomic comparison. JH and ZX participated in some of the phenotyping and genetic diversity analysis. FL, YW, YN, JG, DL, ZL, ZH, YL, and XZ participated in the field investigation and logistic work. QY supervised the study. HH and WQ designed the study and wrote the manuscript. All authors contributed to the article and approved the submitted version.

## Conflict of Interest

The authors declare that the research was conducted in the absence of any commercial or financial relationships that could be construed as a potential conflict of interest.

## Publisher’s Note

All claims expressed in this article are solely those of the authors and do not necessarily represent those of their affiliated organizations, or those of the publisher, the editors and the reviewers. Any product that may be evaluated in this article, or claim that may be made by its manufacturer, is not guaranteed or endorsed by the publisher.

## Footnotes

1
https://ngdc.cncb.ac.cn/gvm/showIndividuals?stype=2&orgId=2

## Supplementary Material

Supplementary Figure 1 Genetic structure and association analysis of Guiping wild rice population (*K* = 6–8). The represented subgroup as same as Figure 2.

Supplementary Figure 2 Haplotype networks analysis of plant architecture related genes *OsPROG1*, *OsLAZY1*, and *OsMOC1* in Guiping wild rice population and 5K dataset. For each gene, left two figures represented phenotypic analysis according to the haplotype, and right figure represented the Haplotype networks.

Supplementary Figure 3 Haplotype networks analysis of other plant architecture related genes *OsTAC1*, *OsLIC1*, *OsDLT*, *OsMOC3*, *OsTB1*, and *OsTAD1* in Guiping wild rice population and 5K dataset. For each gene, left two figures represented phenotypic analysis according to the haplotype, and right figure represented the Haplotype networks.
