# Supplementary material for: Genomic Analysis Provides Insights Into the Plant Architecture Variations in in situ Conserved Chinese Wild Rice (Oryza rufipogon Griff.)
Source: Front Plant Sci. 2022 Jun 27;13:921349. doi: 10.3389/fpls.2022.921349 (PMC9272029; doi:10.3389/fpls.2022.921349)
Supplement: Supplementary file 1 [file Table_1.DOCX]

**Table S1.** Information of primers used in this study.

| **McCouch Locus_ID** | **Chromosome** | **Forward Primer** | **Reverse Primer** | **Expected Marker Length(bp)** | **start** | **end** |
| --- | --- | --- | --- | --- | --- | --- |
| Indel1-4F | 1 | CAGCATACAGTACGCATCATCA | GCTCGTATCTCGATGAGTCCA | 151 | 7806422 | 7806443 |
| Indel1-9F | 1 | TCCCCTCTCCACTTGTCAAC | TTTTTAGTGGCATAGATCCTCTTC | 173 | 20055808 | 20055827 |
| RM10716 | 1 | CCTTCTTGCCGCCAACCTAGTCC | TTTGGCTGACATGACGCCTACG | 320 | 11240059 | 11240080 |
| Indel2-4F | 2 | AGTGTCCCAAGCGAGAAAAC | ATGCACGAGTGAGTGTGAGC | 130 | 7985272 | 7985291 |
| Indel2-3F | 2 | ATAGGGTGGGTGTGCTGAAC | GCACAAAACTGCAGGTCTCC | 183 | 5477492 | 5477511 |
| RM12923 | 2 | AAATGCACAGGCATTCGTAGACC | GAAGAAGTGGATGGAGGACATGG | 217 | 9543381 | 9543401 |
| RM13406 | 2 | AGATCGTAGGGCACGGAGAGG | TGATTAGCGAACAGAGTTGAGACAGC | 266 | 20724329 | 20724349 |
| Indel3-7F | 3 | CTGCACCGGAGAAATTTGAT | CGCATGCAGATGAATAGGTG | 215 | 3399885 | 3399904 |
| Indel3-23F | 3 | TGCAGTTTGTATTCGGGTTG | CGTGGCACATGTGGTTACTT | 191 | 24876098 | 24876117 |
| RM14759 | 3 | GACTCCTTCCTGCTCTCGTTGC | CTGCACCTTTGCACTGAACACC | 196 | 9725791 | 9725836 |
| RM15347 | 3 | GCTACTGCTGCTACTGGAGAAAGG | AGGAACCCTTCTTGAGGTTGG | 271 | 21013878 | 21013898 |
| Indel4-3F | 4 | GGTCGCTGGTTCTTGAATGT | AATGGTTTGACTTCGACCAAA | 170 | 5630637 | 5630656 |
| Indel4-10F | 4 | TCCGTTGATATCGTGCCTAA | GGATGTACAGGTGGATTGAAGG | 149 | 23245996 | 23246015 |
| RM16555 | 4 | GAGATAAGATGAGGCGGAAGAATCG | GCAATGCGTTGTATTTGCAGTGG | 289 | 8682672 | 8682691 |
| RM5424/RM16952 | 4 | TAAAGGTGTCCGACAAGAACACG | GATCGATCTGGAGGATTGAAAGG | 199 | 20608584 | 20608615 |
| Indel5-2F | 5 | ACCTCATCATGCTGAACGTG | TGAGGAACTCCGACTTCTGG | 203 | 3039343 | 3039362 |
| Indel5-9F | 5 | CTGCACTGCTGAAATGGAGA | GCTGGGATATCAACCCTACG | 173 | 20786379 | 20786398 |
| RM18502 | 5 | CGAGTGTGAGCCACGTCATCG | CCGAGCATGGAAAGGAGGAGAGG | 223 | 17145093 | 17145112 |
| Indel6-4F | 6 | CAGTTAACACCAATCCAATCCA | CCAAATGGGCAGTAGTTTGAA | 166 | 8027637 | 8027658 |
| Indel6-9F | 6 | GCGGCTTTGAGAGTTGTTTT | TCGTCATGGTAGCAGAGAGG | 138 | 20643372 | 20643391 |
| RM19725 | 6 | GATCCGAAGATGTGCCAGACAGG | GCCATGGCGAGGAGATTTGG | 255 | 8134068 | 8134111 |
| RM20111 | 6 | GGCAACTCACAAGAGATCCATCC | GGTCATTCTTACCATACCACCTCTCC | 186 | 17752275 | 17752296 |
| Indel7-5F | 7 | TGGTTCTTGGAGCATCTGTG | GTTCCTTCACCAGCGTCATT | 134 | 10766594 | 10766613 |
| Indel7-9F | 7 | TGAATTATTAGCACGCGGTTT | TCCCTCCTTGTCGTCTTTTT | 142 | 20884497 | 20884517 |
| RM21236 | 7 | GTTTGACCGCGGTTTGACTAACG | TTTGAGTGGCATGCAAACAACG | 292 | 6783420 | 6783441 |
| Indel8-3F | 8 | TTTTAAAGCTGCGCCAAAAG | CATAACCGGTAAAGGAGTAGCC | 129 | 5547149 | 5547168 |
| Indel8-8F | 8 | CCCTCCATGTTGTGAGTTCC | TGATTAGATCCAGAAAGGGAGAA | 171 | 18089522 | 18089541 |
| RM22959 | 8 | TACACCACCTGGTTCACCAATCC | CACAAGTGCTGAAGAATCTCACTGG | 192 | 16574837 | 16574857 |
| Indel9-2F | 9 | AAGGAGCATCTTTCTAGCATCG | ACGCCTGGAAGACAATCAAA | 191 | 3048198 | 3048218 |
| Indel9-8F | 9 | GTGCCCGTAATGCTCTCAAT | TGAAAGGTTGATCCTGAACAT | 149 | 18110040 | 18110059 |
| RM23842 | 9 | TATAGGGTGCTACGTGACGATACGG | GCGCAAATCCAGTCAAGTCTCC | 263 | 5543798 | 5543821 |
| Indel10-6F | 10 | AATGACAAGGCCGACGATAG | TATTACCCAGGCCAACCTGT | 153 | 13535591 | 13535610 |
| Indel10-8F | 10 | CTCAGTTGTTGGGGGATGAG | CTTTGGAGATGTGCCAGAGA | 124 | 18592303 | 18592322 |
| RM25375 | 10 | TGTAGCTGCACATCTCCTTCAGC | GCTCATCTCCAAGCTGCAGTCC | 218 | 13207252 | 13207308 |
| Indel11-4F | 11 | TGAGATGTGGCCATTAAGGA | TGGCAAAAGATCTTATATTTACTTCG | 154 | 8380755 | 8380774 |
| Indel11-9F | 11 | TGCAGTACAACACTCAGTTCAAA | CATGTTACGGTACTGGCATCA | 174 | 21476813 | 21476835 |
| RM26319 | 11 | CCACATACGGTGGTCTGACTGG | TAGATCGTGTTTAATGGACGGTCAGG | 271 | 7235165 | 7235200 |
| Indel12-3F | 12 | CGTTCTCTGCACACGCTAAG | CCGTAGGTCAGTTGGTTTCC | 139 | 5495065 | 5495084 |
| Indel12-9F | 12 | AAACCAAAACAAGGCAGTCG | CTCTGAACGGATGACGGATT | 182 | 20450893 | 20450912 |
| RM28107 | 12 | AAAGTACGCCAGAGACTAAGAGAAGG | CAAGACTCACACACAAGACACATAGG | 240 | 16100735 | 16100796 |
