# Supplementary material for: Genomic Analysis Provides Insights Into the Plant Architecture Variations in in situ Conserved Chinese Wild Rice (Oryza rufipogon Griff.)
Source: Front Plant Sci. 2022 Jun 27;13:921349. doi: 10.3389/fpls.2022.921349 (PMC9272029; doi:10.3389/fpls.2022.921349)
Supplement: Supplementary file 2 [file Table_2.DOCX]

**Table S2.** The ABBA-BABA tests of introgression in wild rice population.

| P1 | P2 | P3 | P4 | Dstatistic | Z-score | p-value | f4-ratio | ABBA | BABA |  |
| --- | --- | --- | --- | --- | --- | --- | --- | --- | --- | --- |
| Wild_Rice | cB | wild20 | *Oryza barthii* | 0.052158 | 2.93325 | 0.00335435 | 0.282387 | 22951 | 20675.5 | |
| Wild_Rice | VI_*Aromatic* | wild20 | *Oryza barthii* | 0.0917205 | 4.95621 | 7.19E-07 | 0.468685 | 22476.2 | 18699.6 | |
| cA | XI-1A | wild20 | *Oryza barthii* | 0.0567089 | 2.37453 | 0.0175713 | 0.181171 | 22931.3 | 20470.1 | |
| cA | *Indica*_I | wild20 | *Oryza barthii* | 0.0908017 | 3.79475 | 0.00014779 | 0.277391 | 22635 | 18866.6 | |
| cA | XI-1B | wild20 | *Oryza barthii Oryza barthii* | 0.0668473 | 2.9683 | 0.0029945 | 0.219009 | 23742 | 20766.7 | |
| cA | *Indica*_II | wild20 | *Oryza barthii* | 0.0987891 | 4.3872 | 1.15E-05 | 0.311409 | 23527.4 | 19296.8 | |
| cA | XI-adm | wild20 | *Oryza barthii* | 0.0713645 | 3.27672 | 0.00105022 | 0.23026 | 23480.7 | 20352.6 | |

**Table S3.** Information of 10 plant architecture related genes used for haplotype networks analysis.

| Gene name | Gene_id1 | Gene_id2 | Chr. | Gene_lenth | Range_from | Range_to |
| --- | --- | --- | --- | --- | --- | --- |
| OsPROG1 | LOC_Os07g05900 | Os07g0153600 | chr07 | 895 | 2838194 | 2841089 |
| OsLAZY1 | LOC_Os11g29840 | Os11g0490600 | chr11 | 5523 | 17317918 | 17325441 |
| OsTAC1 | LOC_Os09g35980 | Os09g0529300 | chr09 | 3139 | 20730589 | 20735728 |
| OsLIC1 | LOC_Os06g49080 | Os06g0704300 | chr06 | 3619 | 29737880 | 29743499 |
| OsDLT | LOC_Os06g03710 | Os06g0127800 | chr06 | 3083 | 1464500 | 1469583 |
| OsMOC3 | LOC_Os04g56780 | Os04g0663600 | chr04 | 1050 | 33859374 | 33862424 |
| OsTB1 | LOC_Os03g49880 | Os03g0706500 | chr03 | 1934 | 28427504 | 28431438 |
| OsTAD1 | LOC_Os03g03150 | Os03g0123300 | chr03 | 3572 | 1326450 | 1332022 |
| OsDHD1 | LOC_Os11g47920 | Os11g0706200 | chr11 | 1781 | 28898015 | 28901796 |
| OsMOC1 | LOC_Os06g40780 | Os06g0610350 | chr06 | 3174 | 24312523 | 24317697 |
