# Supplementary figures and images for: Genomic Analysis Provides Insights Into the Plant Architecture Variations in in situ Conserved Chinese Wild Rice (Oryza rufipogon Griff.)
Source: Front Plant Sci. 2022 Jun 27;13:921349. doi: 10.3389/fpls.2022.921349 (PMC9272029; doi:10.3389/fpls.2022.921349)

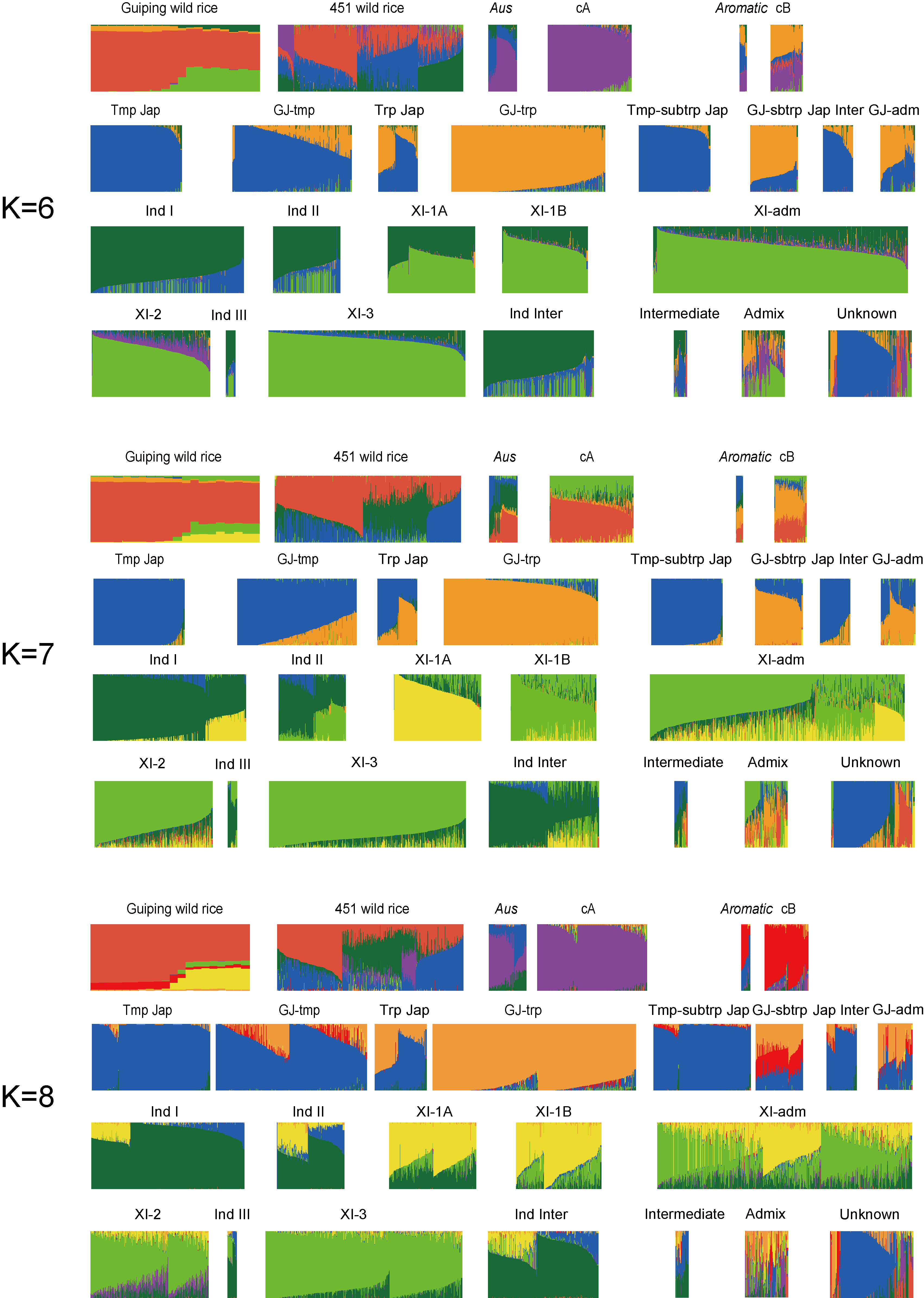

Supplement: Supplementary Figure 1 — Genetic structure and association analysis of Guiping wild rice population (K = 6–8). The represented subgroup as same as Figure 2. [file Image_1.JPEG]

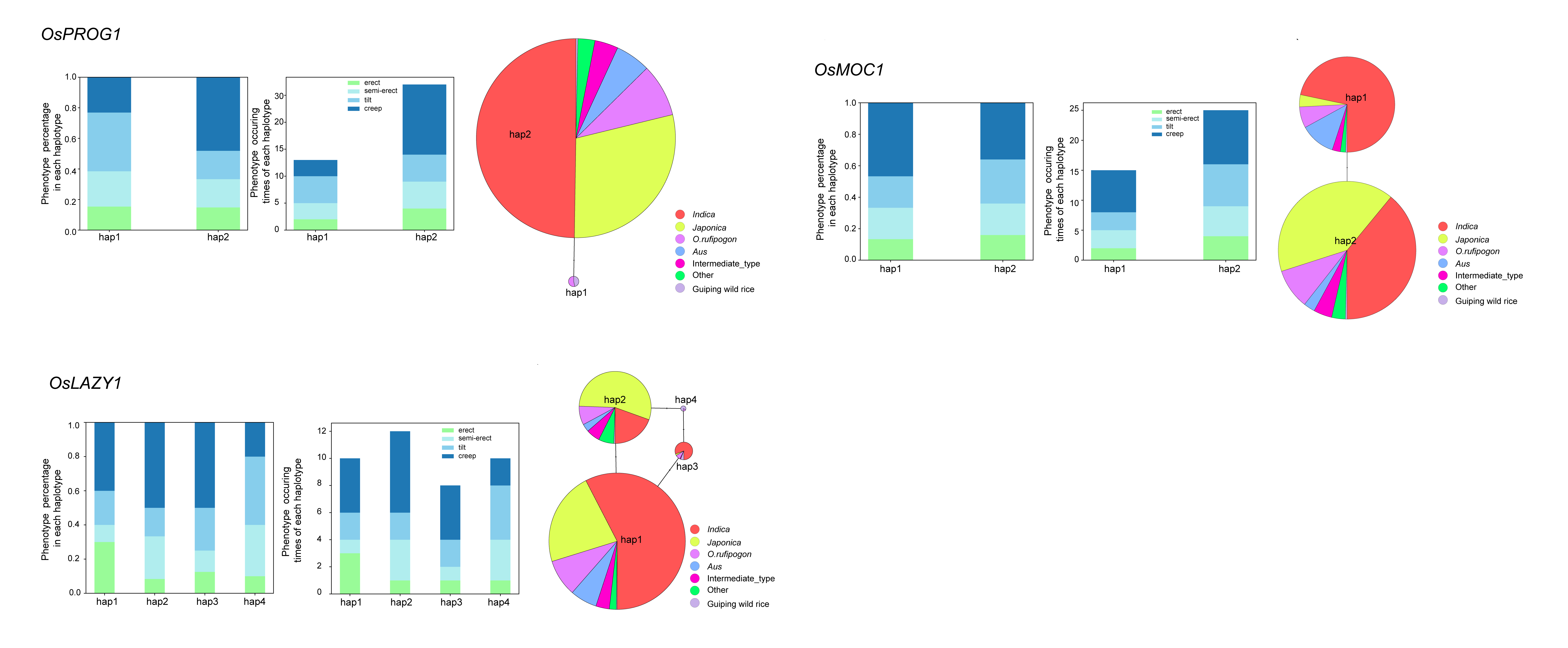

Supplement: Supplementary Figure 2 — Haplotype networks analysis of plant architecture related genes OsPROG1, OsLAZY1, and OsMOC1 in Guiping wild rice population and 5K dataset. For each gene, left two figures represented phenotypic analysis according to the haplotype, and right figure represented the Haplotype networks. [file Image_2.JPEG]
